# The DARE study of relapse prevention in depression: design for a phase 1/2 translational randomised controlled trial involving mindfulness-based cognitive therapy and supported self monitoring

**DOI:** 10.1186/1471-244X-12-3

**Published:** 2012-01-19

**Authors:** Frances Shawyer, Graham N Meadows, Fiona Judd, Paul R Martin, Zindel Segal, Leon Piterman

**Affiliations:** 1 School of Psychology and Psychiatry, Monash University, Clayton Victoria 3800, Australia; 2 Centre for Women's Mental Health, The Royal Women's Hospital, Parkville Victoria 3052 Australia; 3 School of Applied Psychology, Griffith University, Mount Gravatt, Queensland, 4122, Australia; 4 Centre for Addiction and Mental Health, Clarke Division, Toronto, Ontario, Canada, M6B-2H8; 5 Office of the Pro Vice Chancellor, (Berwick & Peninsula), Monash University, Narre Warren Victoria 3805, Australia

**Keywords:** Randomised controlled trial (RCT), Mindfulness-Based Cognitive Therapy (MBCT), Major Depressive Disorder, translational research, health economics

## Abstract

**Abstract:**

**Background**

Depression is a common condition that typically has a relapsing course. Effective interventions targeting relapse have the potential to dramatically reduce the point prevalence of the condition. Mindfulness-based cognitive therapy (MBCT) is a group-based intervention that has shown efficacy in reducing depressive relapse. While trials of MBCT to date have met the core requirements of phase 1 translational research, there is a need now to move to phase 2 translational research - the application of MBCT within real-world settings with a view to informing policy and clinical practice. The aim of this trial is to examine the clinical impact and health economics of MBCT under real-world conditions and where efforts have been made to assess for and prevent resentful demoralization among the control group. Secondary aims of the project involve extending the phase 1 agenda to an examination of the effects of co-morbidity and mechanisms of action.

**Methods/Design**

This study is designed as a prospective, multi-site, single-blind, randomised controlled trial using a group comparison design between involving the intervention, MBCT, and a self-monitoring comparison condition, Depression Relapse Active Monitoring (DRAM). Follow-up is over 2 years. The design of the study indicates recruitment from primary and secondary care of 204 participants who have a history of 3 or more episodes of Major Depression but who are currently well. Measures assessing depressive relapse/recurrence, time to first clinical intervention, treatment expectancy and a range of secondary outcomes and process variables are included. A health economics evaluation will be undertaken to assess the incremental cost of MBCT.

**Discussion**

The results of this trial, including an examination of clinical, functional and health economic outcomes, will be used to assess the role that this treatment approach may have in recommendations for treatment of depression in Australia and elsewhere. If the findings are positive, we expect that this research will consolidate the evidence base to guide the decision to fund MBCT and to seek to promote its availability to those who have experienced at least 3 episodes of depression.

**Trial Registration:**

Australian New Zealand Clinical Trials Registry: ACTRN12607000166471

## Background

The 1 year prevalence of major depressive disorder (MDD) in Australia has consistently been measured above 4% [1,2] and it is commonly a recurrent condition. At least 60% of people who have had one major depressive episode will have another, mostly within 2 years of the index episode [3]. Seventy percent of those who have had two episodes will have a third, and 90% of those with three episodes will have a fourth [4]. Effective interventions targeting relapse, particularly in people with a history of three or more episodes of depression, could dramatically reduce the point prevalence of the condition [5]. Mindfulness-based cognitive therapy (MBCT) is a group-based program that integrates aspects of cognitive behaviour therapy (CBT) with components of a Mindfulness-Based Stress Reduction program [MBSR - [6]]). It was developed specifically with the aim of reducing recurrence rates of MDD by increasing patients' ability to recognize and disengage from depression-related ruminative thought patterns through the cultivation of mindfulness. A recent meta-analysis [7] confirmed a highly significant reduction in relapse for MBCT compared to treatment as usual (TAU) for people with three or more episodes of depression, with effects at least equal to maintenance antidepressant medication. MBCT is now included in the United Kingdom's National Institute of Health and Clinical Excellence (NICE) Guidelines for prevention of depressive recurrence for patients who have experienced three or more episodes of depression [8].

The work reviewed by Chiesa et al [7] could be seen as meeting the core requirements of phase 1 translational research in which the safety and efficacy of treatments for selected volunteers are tested in randomised controlled trials (RCTs) [9]. Although mentioned in the NICE guidelines, MBCT has not been similarly recommended in other countries and its implementation within clinical services is typically not routine. There is a need now to move to phase 2 translational research - the application of MBCT within real-world settings with a view to directly informing policy and clinical practice [9,10]. There are a number of important priorities to consider within a phase 2 agenda for MBCT.

### Advancing the translational research agenda related to MBCT

#### Transportability and effectiveness

MBCT trials to date could be argued as being efficacy studies in which the developers of MBCT either provided the therapy [11,12], or provided direct training [13-15] or direct consultation [16] to the therapists. Hence, a priority for phase 2 translational research is to address the transportability of the program [17] - that is, to what extent can MBCT be successfully adapted and applied in settings away from the original developers. In addition to the general question of replication of MBCT findings, it is unclear what level of training is required to become an effective MBCT facilitator. For instance the Centre for Mindfulness Research and Practice in the School of Psychology, Bangor University offers a Masters course with prerequisites including at least 6-12 months with a personal mindfulness practice and completion of an 8-week mindfulness course. Undoubtedly this makes for a comprehensive and thorough training but the cost and demands on therapists is high when considered against typical training requirements for other brief therapeutic models in regular use (e.g., see http://iptinstitute.com/). The demonstration of positive effects achieved by instructors trained in a briefer and more readily generalisable model could be valuable in establishing the case for transportability of the delivery of MBCT.

#### Antidepressant medication

In considering the general application of MBCT more needs to be known about combination treatment with MBCT and antidepressants [18]. Trials of MBCT to date have typically been structured so as to consider MBCT as an *alternative *to maintenance anti-depressant medication (m-ADM) rather than a complement. This stands in some practical tension with clinical guidelines for treatment of people with 3 or more prior episodes, which generally advise m-ADM for up to 3 years [19]. Only one study to date has examined outcomes for MBCT where participants continue their usual treatment, including m-ADM [16]. At baseline, the majority of participants were taking antidepressant medication with the rate of antidepressant use non-significantly higher in the MBCT group (73%) compared TAU (61%). Of interest, the MBCT relapse rate in this trial was one of the lowest recorded compared to other MBCT groups where anti-depressants was disallowed. Over the course of the follow-up period, the rate of antidepressant use decreased in the MBCT group but not in the TAU group, however, regression to the mean may account for this finding as there were no group differences in the rate of antidepressant medication use at any time point. More detailed research examining the combined role of MBCT and antidepressants is required. Difficulties in interpreting the findings from this study could be seen as an argument for stratification according to m-ADM in a pragmatic study.

#### Longer follow up

To date, the follow-up period in RCTs of MBCT has been limited to around 12 months excepting one 18 month follow-up study [15]. One observational study suggests improvements in depression scores following MBCT are maintained over at least 2 years [20] but its role in relapse prevention has not yet been more rigorously examined over this longer time frame. The vulnerability occasioned by having three or more prior episodes persists over many years [21] so MBCT usefully could be studied for potential protective effect over longer time spans. MBCT involves substantial personal commitment for participants including perhaps significant lifestyle changes in the longer term, so for this reason also, information on enduring benefits is very relevant.

#### Health Economics

Kuyken et al [14] examined the cost effectiveness of MBCT compared to m-ADM. Although not conclusive, this work suggested that MBCT was more expensive than m-ADM over the first 12 months with costs then converging and MBCT becoming cheaper over the subsequent 3 months. The authors speculated that the cost-effectiveness of MBCT may increase over time and recommended examining the cost-effectiveness of MBCT over longer follow-up periods. More generally, later phase translational research questions would be served by a health economics study and analysis taking into account local service context, real-world conditions including combined treatment with anti-depressant medication and multiple economic perspectives.

#### Continuing phase 1 agenda

##### Mechanisms of action

Although a number mediation analyses have been published in relation to MBCT, the mechanisms of action remain unclear. Mediation studies often have been small and hence with low power, or limited by being observational in nature [13,20,22-24]. So to consider some possible mediation factors needing further examination:

• Time spent practicing mindfulness meditation correlates with reductions in mood disturbance and relapse but how much practice is required for therapeutic benefit in MBCT remains unsettled [13,20,25].

• Evidence for the role of mindfulness scores in mediating depressive relapse outcomes in MBCT is inconsistent [23,26].

• There has been some support for proposed interrelationships between mindfulness, rumination and depression in an observational study [20] and from a small RCT [24] but the findings await replication in a larger controlled design.

To date the evidence supporting a central mediation role for mindfulness and rumination mediating outcomes in terms of depression and especially depressive relapse is, at best, patchy.

##### Comorbidity

Anxiety disorders are frequently associated with depression and, importantly, are a significant risk factor for the development of depression [27]. Relaxation, although not a primary goal of the mindfulness practices taught in MBCT, is commonly experienced through many of the exercises [28] and learning of relaxation skills may assist participants with anxiety disorders. Moreover, the process of rumination may be similar in anxiety and depression, although the content may differ [29]. A meta-analysis of previous studies suggests that mindfulness-based interventions may have a positive impact on anxiety [30]. However, the mediation role of anxiety in long-term management of depression with MBCT has not yet been examined.

##### Need to prevent and assess for resentful demoralisation

"Resentful demoralisation" is a well-recognised bias that may arise in control group participants whereby the belief that they are not receiving a desirable treatment negatively affects their attitude and behaviour, and as a consequence, the outcome results. Thus, a criticism that that has been leveled at much of the work so far with MBCT is that the promising findings in favour of MBCT may be a reflection of relative deterioration in the TAU group rather than improvement in the MBCT group [31]. As noted by Everitt and Wessely [32], participants entering a trial "are likely to believe that the new therapy is about to solve all their problems. Why else would it be featuring in the trial?" (p. 16). If a participant is then not provided with this promising new therapy, he or she may feel disappointed and have negative expectations of their ongoing involvement. If such negative expectations are present, the seemingly positive results for the experimental treatment may in fact be accounted for by the negative effects in the control. The control condition in this scenario possibly acquires properties of a nocebo [33]. Given that trial participants in MBCT studies are typically highly vulnerable to depression and therefore negative thinking, this effect could be important to consider in study design.

#### Aims of trial

Within the parameters of phase 2 translational research, the aim of this effectiveness study is to examine the clinical impact and health economics of MBCT where efforts are made to assess for and prevent resentful demoralization and under real-world conditions - within a population representative of the intended target audience, that is, people with a history of depression regardless of their antidepressant regimen. For a long-term preventive intervention evidence of a longer duration of effect would be more desirable than the 12-18 month periods so far examined and 2-year follow-up is planned in this study. The portability of MBCT training has not been assessed and this project will investigate outcomes from a novel training program using instructors who may not have a long-standing and deep knowledge of the intervention provided.

**Figure 1:**
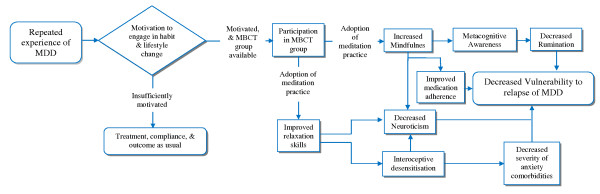
**Proposed mechanisms for MBCT**.

The secondary aim of the project is to provide for a detailed examination of the mechanisms by which this form of therapy may prevent depressive relapse, with this examination structured in a way so as also to progress the understanding of the clinical applicability of MBCT as a treatment for relapse prevention in depression. MBCT may act to reduce relapse through a number of mechanisms outlined in the model shown in Figure 1. According to this model, the two primary mediators for outcome in terms of reduced depressive relapse are 1) increased mindfulness and 2) improved relaxation skills which occur as the result of adopting a meditation practice. We have postulated that these factors may have positive downstream effects on other secondary mediating factors such as rumination, neuroticism, medication adherence and anxiety. Although it will not be practicable to explore validation of this causal nexus fully, we will use it to guide our choice of measures through the research plan that follows.

## Methods/Design

### Research design

This study is a prospective, multi-site, single (rater)-blind, RCT using a group comparison design between involving the intervention, MBCT, and a comparison condition, Depression Relapse Active Monitoring (DRAM) with follow-up over 2 years. Both interventions are provided in addition to TAU. All participants receive DRAM with half the sample randomised to additionally receive MBCT. Participants continue their usual treatment during the course of their participation in the trial. We avoid any attempts to standardise routine clinical care including medication beyond the recommendations of DRAM. The study is being conducted in compliance with the Helsinki Declaration and has been approved by the following governing ethics committees: Alfred Hospital Ethics Committee (280/07); Barwon Health Research and Ethics Advisory Committee (07/86); Monash University Standing Committee on Ethics in Research Involving Humans (CF07/1275-2007/019MCC; CF08/1031-2008000509); Peninsula Health Human Research & Ethics Committee (2007-53); Southern Health Human Research Ethics Committee (07056B); and The Melbourne Clinic Research Ethics Committee (189). Written informed consent is required for all participants.

### The Interventions

#### MBCT

##### Description

MBCT is a manualised program delivered by an instructor in 8 weekly 2-hour group training sessions involving up to 10 clients; optional 3-monthly 'booster sessions' are also delivered. Sessions incorporate mindfulness practices including meditation and also CBT exercises [28]. Homework for participants includes formal daily meditation practices and exercises for the development of mindful awareness within everyday activity.

##### Therapist training

MBCT instructors are selected from a locally-developed MBCT professional training program headed by GM, who was trained by one of the developers of MBCT (ZS). The training program is, we believe, uniquely geared toward efficiently training experienced mental-health clinicians who do not necessarily have any background or knowledge of meditation to be MBCT instructors. Thus, positive results from this trial favouring MBCT would suggest that it can be effectively provided by instructors who may not have a long-standing and deep knowledge of the intervention and who are trained by someone outside of the original developers of the intervention. This will add further evidence in terms of the important issue of the transportability and generalisability of treatment [7].

The training program involves an initial 8-week course that is a variation of the MBCT therapy group but adapted for the training situation, with some variation to the exercises so as to make them more suitable for experiential participation and learning by therapists rather than patients with depression. Since it is not assumed that trainees already have a meditation practice, following the 8-week course, there is a break for a minimum period of 1 month so that participants can consolidate their personal practice and associated learning. The program then ends with an intensive teacher development retreat over 3 full days which continues to emphasise personal practice as well as concentrated and closely coached practice in leading meditations, discussions and other aspects of the course (total of 44 hours training).

##### Quality control

MBCT sessions are video-taped with the patients' permission (camera visual field focused on clinician) for the purposes of supervision and assessing treatment fidelity. An independent audit of the therapy will be undertaken across the therapy groups using two sessions randomly selected from each group. Sessions will be rated by a clinical psychologist with training and experience in MBCT using the MBCT-Adherence Scale [34].

#### Depression Relapse Active Monitoring (DRAM)

To reduce the discrepancy between groups in treatment expectation and thus mitigate against the risk of triggering resentful demoralisation, we developed a comparison condition called "Depression Relapse Active Monitoring" (DRAM) as an alternative to a TAU-only control. DRAM comprises training on the management of depression through the monthly self-monitoring that forms part of the research assessments. To support DRAM, participants are provided with a manual (also available on the project website) and a wallet-sized card which has self-monitoring items, emergency phone numbers and project contact and web details printed on it. Training in DRAM is provided by research assistants during the intake assessment interview. By being able to fairly present the project as having a potential benefit for all participants, we expect to not only reduce the potential for resentful demoralization but also selection bias. The project title, marketing name and marketing materials have also been developed so as to reflect both conditions in an unbiased manner.

### Power analysis

A power analysis was conducted using estimates of annual relapse rates obtained from the literature. Systematic reviews and meta analyses were mainly utilised including: no treatment (or placebo) only [35-38]; psychological treatment alone; effect in recurrent depression of medication alone [35,37,38]; and combined psychological and pharmacotherapy [39,40]. From these sources, anticipated relapse rates over a 2-year period were: no treatment, 56%; MBCT alone, 31%; medication alone, 31%; and MBCT and medication together, 15%. Based on findings from recruitment in our pilot study, we assumed that 70% of the sample would be taking at least some reasonably effective dose of medication, and 30% would not. Applying the subgroup specific odds ratios suggested the following relapse rates as likely over a 26-month period: MBCT group 21.5%; and DRAM group 41.7%. A power analysis showed that to detect this difference using an uncorrected chi-squared statistic at 80% power and α = 0.05 would require a sample size of 82 participants to complete the full 26 months in each of the MBCT and DRAM groups [41]. Further, assuming a dropout rate of 20% led to a recruitment target of 102 participants for each group.

### Participants

#### Recruitment

Participants are recruited from private and public community health services and through notices placed in local media in metropolitan Melbourne and the regional city of Geelong, located in the state of Victoria, Australia. Also targeted are public mental health services across multiple sites and ECT clinics where the more severely affected individuals are likely to be treated as, from a health economics perspective, this group is likely to have the greatest impact on costs.

#### Selection criteria

Inclusion criteria include meeting DSM-IV criteria for three previous episodes of MDD [DSM-IV diagnosis of either MDD (Recurrent) or Bipolar Disorder I or II], aged between 18-75 years, and able to speak and read English fluently. Current use of medication, including antidepressants and mood stabilisers, is allowed. Exclusion criteria include: current episode of MDD; current symptoms of a psychotic disorder, or a past diagnosis of a psychotic disorder where the treating clinician believes the therapy may be contraindicated; current significant eating disorder or obsessive-compulsive disorder; organic mental disorder or pervasive developmental delay; current borderline or antisocial personality disorder; current alcohol or drug dependency other than tobacco; current benzodiazepine intake of more than 20 mg diazepam equivalent; and inability to give informed consent.

#### Randomisation

Assignment of individual participants to MBCT + DRAM or DRAM alone is undertaken by the study statistician using a minimization routine balancing numbers in groups as randomisation proceeds [42]. The statistician works independently of staff involved in the recruitment, assessment and management of participants in the study. To guard against confounding by major differences in other treatment exposures, randomisation is stratified by: medication (currently taking antidepressants and/or mood stabilisers: yes/no); site of referral (primary care, secondary care, specialist clinic): diagnosis (bipolar disorder: yes/no); and gender.

#### Retention

Given the 2-year follow-up proposed for this study, and the requirement of monthly assessments, attention to retention strategies is crucial to the success of the trial as completeness of follow-up has a significant effect on the quality and validity of the analysis. Strategies we implement to limit attrition include: minimising response burden as far as possible such as having flexibility in how assessments can be completed; employing staff experienced in communicating with research participants; establishing a project name ("DARE - Depression Awareness Recovery Effectiveness") and identity (professionally designed project logo, website, wallet card with key contact numbers); provision of reimbursement that acknowledges the participants' time and effort; and greeting cards and quarterly newsletters to maintain positive contact between assessments.

### Measures

#### Diagnosis and eligibility

##### Composite International Diagnostic Interview 2.1 Auto Lifetime version (CIDI-LT)

Diagnostic inclusions and exclusions are assessed using the appropriate sections of the Composite International Diagnostic Interview 2.1 Auto Lifetime version (CIDI-LT). The CIDI 2.1 is a highly standardised instrument designed to be administered by lay interviewers. The CIDI 2.1 was used in preference to the more recent World Health Organisation CIDI (WHO CIDI) because the latter is not available in a 12-month form and the former had stronger evidence supporting its validity [43,44]. To minimise participant burden, in this design exclusion criteria related to psychosis, personality disorder, organic mental disorder or pervasive developmental delay are not formally assessed. Instead, reliance is placed on the treating clinician to screen out ineligible candidates.

#### Primary outcome: relapse/recurrence of MDD

##### Composite International Diagnostic Instrument 2.1 Auto 12 month version (CIDI-12)

Episodes of depression are assessed retrospectively during a face-to-face interview using the CIDI Auto 2.1 12 month affective disorders modules. Several questions from the WHO CIDI are added to give more precise details of episodes including number of episodes (where there was at least 2 weeks in remission between episodes), the month that the first/only depressive episode commenced, and total number of days depressed.

##### Patient Health Questionnaire

In the study the Patient Health Questionnaire-2 [PHQ-2 - [45]] and the PHQ-9 [46] are used to monitor for the presence and severity of depressive symptoms each month. The PHQ-2 is a screening tool for depression that comprises two questions addressing anhedonia and depressed mood with frequency ratings 0 (not at all) to 3 (nearly every day). The PHQ-9 comprises the nine criteria upon which the diagnosis of DSM-IV depressive disorders is based. Both instruments have been found to be reliable and valid [45,46]. As part of DRAM, participants are trained on how to complete the PHQ-2 and PHQ-9 then prompted monthly to self-monitor via their preferred medium of SMS, telephone or internet. To facilitate monitoring, the PHQ-2 is provided on a wallet card which also has their project ID and password for online completion. Participants reporting scores ≥3 are directed to complete the PHQ-9 and encouraged to seek appropriate assistance using automated feedback. The full PHQ-9 is administered every 3 months regardless of PHQ-2 score.

##### Time to first clinical intervention

We expect an active property of DRAM in prompting participants to seek help for depressive symptoms at an earlier stage than would occur without this self-monitoring intervention. Thus, although providing some control for treatment expectation and ethically optimising the design, this intervention may reduce the between-group differences with respect to depressive relapse so that time to first clinical intervention is an important adjunctive measure. The General-practice Users' Perceived-need Inventory (GUPI) short form [47] is a three-item measure of perceived need for mental health care that was slightly modified to assess whether there had been a clinical intervention in the past 3 months, and if so, when this was initiated. Study design also includes tracking of medication changes over the course of the project with onset of an antidepressant or increase in dose assumed to signal an incipient relapse.

#### Patient expectations

##### Credibility/Expectancy Questionnaire

The Credibility/Expectancy Questionnaire [CEQ - [48]]) is used to assess therapy credibility and client expectancy for improvement, as potential confounds for outcome. The CEQ has high internal consistency and good test-retest reliability and has been shown to discriminate between different treatment rationales [48]. The instructions for the CEQ are modified so as to be appropriate for participants in both groups.

##### Belief about relapse

Belief about relapse is assessed by asking participants to indicate how likely it was that they would experience depression again in the next year on a fully anchored scale from "very unlikely" (score 1) to "very likely" (score 5).

#### Secondary outcomes and process measures

A range of measures are included to examine the factors shown in Figure 1 in terms of both outcome and mediation. Measures include the State-Trait Anxiety Inventory ([STAI - [49]]), International Personality Item Pool Neuroticism and Openness to experience subscales [IPIP - [50,51]]), Five Facet Mindfulness Questionnaire [FFMQ - [52]]), the Ruminative Response Scale [RRS - [53]]), the Hill-Bone Medication Taking Scale [54] and locally-developed measures of mindfulness practice and medication adherence. Outcome in relation to work and social functioning is assessed using the Work and Social Adjustment Scale [WSAS - [55]]) and health-related quality of life using the Assessment of Quality of Life instrument [AQoL -[56]]).

#### Economic analysis

Service utilisation by self-report is assessed with instrumentation used in the most recent (2007) Australian National Survey of Mental Health and Wellbeing. This instrument was used in the first national survey in 1997, but has been updated to take into account identified deficiencies in the earlier instrument [57]. It includes some elements of the service utilisation section of the WHO CIDI [44]. It also contains questions relating to levels of perceived need for mental health care and so provides a consumer appraisal of the relative adequacy of overall service provision [58-60]. As the instrument was prepared drawing on a national expert group with support of the Australian Bureau of Statistics, this likely represents the best developed instrument for collection of service utilisation data in Australia.

In order to estimate costs associated with lost productivity, every 3 months participants in the study are asked to record the number of days they had been absent from their usual work or occupation due to illness or disability. Also collected is their account of how many of these days were due to depression or related problems with their mental health. Employment status and, for those working, current occupational status are rated using the *Australian and New Zealand Standard Classification of Occupations *[61]. The AQoL will also be used to assess the cost-utility of MBCT in terms of quality-adjusted life years (QALYs).

### Procedure

#### Assessments

Experience from pilot work [62] indicated that the primary reason for exclusion from that study was failure to meet the selection criteria related to MDD. Referrals to the project are therefore screened over the phone using a brief questionnaire based on key questions from the Depression module of the Lifetime version of the CIDI 2.1. Potentially eligible candidates then are invited to complete an intake assessment to assess eligibility and collect baseline data. Following the intake assessment, eligible participants are randomised in cohorts to treatment condition. For participants in both treatment conditions, PHQ assessments are completed at intake and then at monthly intervals from the fourth week of the MBCT program conducted for their cohort. The CIDI 2.1 12 month version, Service Utilization, Hill-Bone Medication Taking Scale, IPPI, CEQ and Beliefs about Relapse are completed at 12 months and 24 months following the conclusion of the MBCT program. The CEQ is also administered at the fourth and eighth week of the MBCT program and Beliefs about Relapse at 8 weeks. The remaining self-report assessments are administered 3 monthly.

#### Blindness

Considerable efforts are made in this design to maintain the blindness of raters for the yearly face-to-face assessments including: written and verbal reminders to participants; a five-minute videotaped explanation of blindness by the principal investigator (GM) shown immediately prior to the 12-month assessment; and a reminder sign placed prominently in front of the participant during the course of the interview. Raters are asked to classify participants into a treatment condition together with any specific reasons for their guess and an estimate of their level of certainty. Breaches in rater blindness are addressed by changing the rater where possible.

### Statistical analysis

#### Primary outcomes

Intention-to-treat (ITT) analysis will be the primary analytic framework. Groups will be compared using chi-square or Fisher's exact test for proportions and *t*-test or Mann-Whitney-test for continuous variables. Time to relapse/recurrence of depression will be examined using the Kaplan-Meier survival analysis. The Cox proportional hazards regression analysis will be used to examine the effect of covariates such as treatment expectations on time to relapse/recurrence. Per protocol (PP) analyses will also be undertaken. As in previous trials, at least four sessions will be considered as the minimal treatment dose. However, as the MBCT program is sequential and participants are strongly encouraged to attend all sessions, or at least miss no more than one [63], we will also examine outcomes for people who attended the recommended dose of therapy, being 7-8 sessions.

#### Secondary outcomes and mediation

Secondary outcomes will be examined using repeated-measures Analysis of Variance (ANOVA) with further analyses to explore mediation [64,65].

#### Economic evaluation

The incremental cost of MBCT is the cost of treatment less any savings in health service use. Results of incremental cost-effectiveness analysis will be subject to extensive probabilistic sensitivity analysis [66] using @Risk software, which allows the results to be presented as cost-effectiveness acceptability curves [67]. The cost-utility of MBCT + DRAM compared to DRAM alone will be compared using repeated-measures ANOVAs.

As recommended by Hollis and Campbell [68], sensitivity analyses will be undertaken across these analyses to examine the potential influence of the missing data using various forms of imputation. Where appropriate, given the design of the study, multilevel modelling will be employed [69].

## Discussion

This study is a 'real-world' examination of MBCT as an intervention designed to prevent depressive relapse. Set within a phase 2 translational research framework, its findings will assist in determining the role that MBCT can play in routine clinical practice by examining whether MBCT is an effective intervention for people with a history of three or more depressive episodes who are undertaking their usual treatment including antidepressants, in a setting outside that of the initial developing group, and across multiple sites. The sample size, which is large relative to previously published MBCT trials, will allow for a secondary outcome and mediation analysis and so provide further knowledge regarding the mechanisms by which MBCT may operate to prevent depressive relapse, both alone and in combination with pharmacotherapy. The training for therapists delivering MBCT in the trial is a novel MBCT professional training program that is relatively brief and does not assume any background knowledge of meditation on the part of trainees. This professional training program is potentially an efficient and replicable way of providing comprehensive training in the instruction of MBCT to enable more rapid dissemination in the community. Positive results from this trial not only would have implications for ease of translation into the community and its transportability to new settings but also for extending our understanding on how much training is necessary for the effective delivery of MBCT.

The need to balance competing questions of interest is an important consideration in the design of RCTs. For example, the recommended comparator for a health economic analysis is a TAU control [70], while an active alternative treatment is likely to provide the most information for studies investigating mechanisms of action [e.g., [71]]. As an effectiveness trial, a health economic analysis is an important aspect of the present study so favouring the selection of a TAU control in the design. However, we had the additional need to control for the potential bias of resentful demoralization and so the self-monitoring intervention DRAM was introduced as an addition to TAU. This is likely to mean that episodes of depression are picked up and treated earlier than would be the case without such monitoring and possibly lead to a relative weakening of the between-group effects for standard symptom measures [72]. Nevertheless, we predict that the impetus to access treatment prompted by DRAM will, if anything, increase between-group differences in the health economic evaluation and so this will form a critical focus for examination. In addition, with respect to relapse outcomes, substantial reliance will be placed on measures relating to time to clinical intervention.

Historically, health care systems have been heavily oriented to the treatment of illness, rather than to its prevention, resulting in costs that have been described as spiralling out of control [73]. The argument has been put forward that where preventative and especially "low tech" interventions such as mindfulness-based interventions are available, priority should be given to exploring how best to implement them for greatest impact [73]. The results of this trial, including an examination of clinical, functional and health economic outcomes, will be used to assess the role that this treatment approach may have in recommendations for treatment of depression in Australia and elsewhere. We expect that the findings from this research will consolidate the evidence-base to guide the decision to fund MBCT and to seek to promote its availability to those who have experienced at least three episodes of depression.

## Abbreviations

ANOVA: Analysis of Variance; AQoL: Assessment of Quality of Life; CBT: Cognitive Behaviour Therapy; CEQ: Credibility/Expectancy Questionnaire; CIDI: Composite International Diagnostic Interview; DARE: Depression Awareness Recovery Effectiveness; DRAM: Depression Relapse Active Monitoring; DSM-IV: Diagnostic and Statistical Manual of Mental Disorders, Fourth Edition; ECT: Electroconvulsive Therapy; FFMQ: Five Facet Mindfulness Questionnaire; GUPI: General-practice Users' Perceived-need Inventory; IPIP: International Personality Item Pool; ITT: Intention to treat; LT: Lifetime; m-ADM: Maintenance Anti-Depressant Medication; MBCT: Mindfulness-Based Cognitive Therapy; MBSR: Mindfulness-Based Stress Reduction; MDD: Major Depressive Disorder; NICE: National Institute of Health and Clinical Excellence; PHQ: Patient Health Questionnaire; PP: Per Protocol; QALY: Quality-Adjusted Life Year; RCT: Randomised Controlled Trial; RRS: Ruminative Response Scale; SMS: Short Message Service; STAI: State Trait Anxiety Inventory; TAU: Treatment As Usual; WHO: World Health Organisation; WSAS: Work and Social Adjustment Scale.

## Competing interests

The authors declare that they have no competing interests.

## Authors' contributions

GM, FJ, PM, LP and ZS conceived and designed the study and obtained funding. FS is the project manager. FS and GM drafted this paper with contributions from all the authors which was then added to and modified by PM. All authors read and approved the final manuscript.

## Authors' information

^1^Southern Synergy, School of Psychology and Psychiatry, Notting Hill Campus, Monash University, c/- Wellington Road, Clayton Victoria 3800, Australia. ^2^Centre for Women's Mental Health, The Royal Women's Hospital, Parkville Victoria 3052, Australia. ^3^School of Applied Psychology, Griffith University, Mount Gravatt Queensland 4122, Australia. ^4^Centre for Addiction and Mental Health, Clarke Division, Toronto Ontario, Canada, M6B-2H8. ^5^Office of the Pro Vice Chancellor, (Berwick & Peninsula), Monash University, PO Box 1071, Narre Warren Victoria 3805, Australia.

## Pre-publication history

The pre-publication history for this paper can be accessed here:

http://www.biomedcentral.com/1471-244X/12/3/prepub
